# *In vivo *kinetics of transcription initiation of the lar promoter in *Escherichia coli*. Evidence for a sequential mechanism with two rate-limiting steps

**DOI:** 10.1186/1752-0509-5-149

**Published:** 2011-09-25

**Authors:** Meenakshisundaram Kandhavelu, Henrik Mannerström, Abhishekh Gupta, Antti Häkkinen, Jason Lloyd-Price, Olli Yli-Harja, Andre S Ribeiro

**Affiliations:** 1 Laboratory of Biosystem Dynamics, Computational Systems Biology Research Group, Department of Signal Processing, Tampere University of Technology, 33101 Tampere, Finland; 2 Institute for Systems Biology, 1441N 34th St, Seattle, WA, 98103-8904, USA

## Abstract

**Background:**

In *Escherichia coli *the mean and cell-to-cell diversity in RNA numbers of different genes vary widely. This is likely due to different kinetics of transcription initiation, a complex process with multiple rate-limiting steps that affect RNA production.

**Results:**

We measured the *in vivo *kinetics of production of individual RNA molecules under the control of the lar promoter in *E. coli*. From the analysis of the distributions of intervals between transcription events in the regimes of weak and medium induction, we find that the process of transcription initiation of this promoter involves a sequential mechanism with two main rate-limiting steps, each lasting hundreds of seconds. Both steps become faster with increasing induction by IPTG and Arabinose.

**Conclusions:**

The two rate-limiting steps in initiation are found to be important regulators of the dynamics of RNA production under the control of the lar promoter in the regimes of weak and medium induction. Variability in the intervals between consecutive RNA productions is much lower than if there was only one rate-limiting step with a duration following an exponential distribution. The methodology proposed here to analyze the *in vivo *dynamics of transcription may be applicable at a genome-wide scale and provide valuable insight into the dynamics of prokaryotic genetic networks.

## Background

Gene expression is inherently stochastic and most RNA molecules exist in very low copy numbers in *Escherichia coli *[1]. The phenotype of these cells depends strongly on how many RNA molecules of each gene are produced [2], when they are produced, and how their numbers fluctuate in time, especially because protein numbers generally follow the RNA numbers [3,4]. This suggests that for the phenotype to be robust and thus predictable, bacteria may need to control fluctuations in some RNAs numbers, especially of weakly expressed genes.

RNA numbers depend on the kinetics if its production and degradation. A genome wide study of degradation rates of RNA molecules in *E. coli *concluded that while there is a wide range of degradation rates, it is the transcription rate that determines mRNA steady-state levels [4]. Differences in RNA half-lives may have other roles, such as the regulation of transient changes in abundance in response to environmental stress or cell cycle [4]. Further, while several sequence dependent events can take place in elongation that affect mean and fluctuations in RNA numbers [5], apart from premature terminations, they only have tangible consequences if multiple RNA polymerases are on the template simultaneously. This only occurs for strongly expressed genes and thus the dynamics of transcription initiation should be the key determinant of the dynamics of RNA numbers for weakly expressed genes.

The mean rate of transcription of a gene is mostly determined by the promoter sequence as well as by the present concentrations of possible activator and repressor molecules. In bacteria, the process of transcription initiation at the promoter region includes diffusion of the RNA polymerase (RNAp) along the template until reaching a transcription start site (TSS), DNA bending and loading in the active site of the RNAp, DNA unwinding and positioning in the TSS, loading of the NT strand, and assembly of the clamp/jaw on downstream DNA [6]. After this sequence of events, the RNAp can elongate along the DNA and assemble the RNA strand. At the termination sequence, the RNAp and a single-stranded RNA are released.

The durations of the rate-limiting steps in initiation vary widely between promoters, even when the sequences only differ slightly [7], as well as with temperature [8] and concentration of Mg^2+ ^and other metabolites [9]. *In vitro *studies of the kinetics of the lac-UV5 promoter in *E. coli *suggest that its initiation involves up to three rate-limiting steps: formation of a closed complex (RPc), isomerization (forming the RPi complex), and formation of the open complex, RPo [8,10,11]. Isomerization is only rate-limiting for temperatures below 20C.

The initiation mechanism is dynamically complex as it involves, e.g., uni-dimensional diffusion of the RNAp on the DNA template and conformational changes of the RNAp and template [12,13]. So far, no measurements exist of the distribution of the duration of these events, and the existing information on the kinetics derives solely from *in vitro *estimations of mean durations. A detailed model [11] of the likely common sequence of events is shown in (1). R stands for RNAp, P stands for promoter DNA, RP stands for the complex of R bound to P, while RPc and RPo stand for the closed and open complexes, respectively. I_1 _to I_3 _are intermediates of the isomerization step. The last step in (1) competes with abortive initiation [14]. Also shown in (1) are the expected speeds of the steps (in the forward direction) given results from *in vitro *measurements on a few promoters [11]:

(1) $$\begin{array}{c} R+P\underset{}{\overset{slow}{↔}}RP\underset{}{\overset{rapid}{↔}}RP_{c}\underset{}{\overset{rapid}{↔}}I_{1}\\ \;\underset{}{\overset{slow}{↔}}I_{2}\underset{}{\overset{rapid}{↔}}I_{3}\underset{}{\overset{rapid}{↔}}RP_{o}\overset{rapid}{\underset{}{\to }}RP_{init} \end{array}$$

All steps in (1), except for the last one, are reversible [13]. *In vitro *studies suggest that the unwinding of promoter DNA, which occurs early in the open complex formation [15] is a slow process compared with the time for the RNAp to diffuse along the template and find a TSS [12]. A simplified model of (1) is shown in (2), showing only the rate-limiting steps [12,13], by packing the fast steps into the three steps known to be slow in some promoters (reversibility not represented):

(2) $$R+P^{{}_{lac-UV5}}\to R{P_{c}}^{{}^{{}_{lac-UV5}}}\to RP_{o}^{{}^{{}_{lac-UV5}}}\to RP_{init}^{{}^{{}_{lac-UV5}}}$$

Let t(RP_c_) be the duration of the closed complex formation (first step in (2)), which includes the time for the RNAp to find the TSS. Also, let t(RP_o_) be the duration of the open complex formation (second step in (2)), and let t(RP_cl_) be the time for RNA chain elongation initiation and promoter clearance (third step in (2)). Finally, let t_pt _be the time to start a productive transcription, equal to the sum of t(RP_c_), t(RP_o_) and t(RP_cl_). *In vitro *measurements of the kinetics of the lac promoter and variants, such as lar, indicate that t_pt _is of the order of 10-1000 seconds, depending on the concentrations of inducers and environmental factors such as temperature.

The *in vivo *kinetics of the steps in (2), as well as the distribution of durations of intervals between initiation events, has not been characterized for any promoter [11]. This distribution is likely a determining factor of the strength of fluctuations in RNA numbers [16]. A recent study using a delayed stochastic model of gene expression suggests that, by regulating the kinetics of the closed and open complex formations, it is possible to regulate both mean and fluctuations in RNA numbers independently [17]. This is relevant since the kinetics of these steps varies with sequence, environmental factors such as temperature, and concentrations of repressor and activator molecules [12]. In general, the binding of a repressor to the promoter significantly increases the duration of the closed complex formation, usually by reducing the probability that an RNAp will find the TSS (e.g. by blocking diffusion on the template) [7,12]. Activators tend to have more complex effects, affecting the mean duration of both closed and open complex formations [7,12].

Recently, a method was developed in *E. coli *to tag mRNA molecules *in vivo *with MS2d-GFP proteins that allows their detection shortly after being produced (Golding et al, 2005). Expression of the target RNA is controlled by the lar promoter (also named lac/ara) [7]. Individual transcription events are detectable and the behaviour is similar to that of the unlabeled system [18,19]. Using this method, we measured intervals between consecutive productions of RNA molecules under the control of lar, under weak and medium induction, which have not been previously measured.

The kinetics of transcription initiation of the lar promoter, as well as of several variants, have been studied *in vitro *[7]. The sequence of the lar promoter and differences from the original lac promoter are described in detail in [7,20]. Its expression is activated by Arabinose and IPTG. *In vitro*, the time between productions of consecutive RNA molecules is approximately 6000 s when not induced, 2500 s when induced by IPTG alone, 800 s when pre-incubated with Arabinose alone, and 50 s when induced with both IPTG and Arabinose [7]. Recent *in vivo *measurements suggest that the kinetics of transcription differs from *in vitro *conditions. For maximum induction, *in vivo*, only 4 RNAs are produced on average in 1 hour [18].

Here, we report *in vivo *measurements of intervals between RNA production events, in the regimes of weak and medium induction. From the distributions of intervals, we derive number of steps and their duration, necessary to describe the measured distributions, assuming that each step's duration follows an exponential distribution. The method proposed here is applicable to study the kinetics of initiation of a wide range of promoters in *E. coli *and, as such, may provide new genome-wide knowledge on the dynamics of transcription initiation in prokaryotes.

## Results

We measured the dynamics of transcript production for weak, medium and full induction of the lar promoter (see Methods and Additional File 1). Each cell produced 0.7 RNA/h on average when weakly induced, and 1.7 RNA/h under medium induction. Under maximum induction, the average production was 4 RNA/h. These averages include cells that did not produce any RNA molecule during the observations.

The difference in the mean rate of production of mRNAs between weak, medium and high induction levels was confirmed with qPCR (Additional File 2). Our measurements of mean production rates agree with those reported in [18] using the same technique and conditions, for each induction strength.

**Figure 1:**
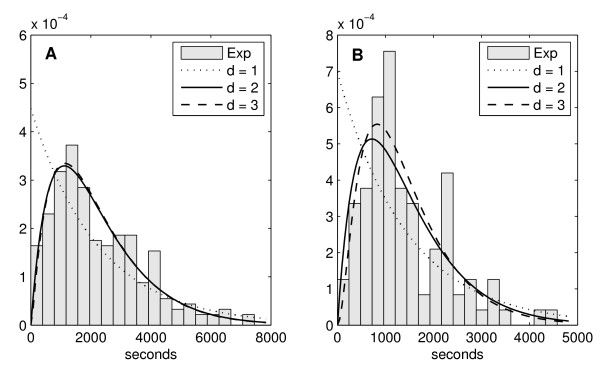
**Histogram of the measured intervals superimposed with the probability density functions of the models**. Distributions of intervals between consecutive transcription events for weak (left) and medium (right) inductions. Each bar is 180 s. Measurement time is 2 hours (measured every 60 s). (A) mean of measured intervals is 2233 s and standard deviation is 1506 s (data from 233 intervals extracted from 283 cells). (B) mean of measured intervals is 1433 s and standard deviation is 1243 s (data from 99 intervals extracted from 40 cells). The histograms of measured intervals are superimposed with probability density functions of models with 1, 2 and 3 steps that best fit the data. Dotted line: 1-step model, solid line: 2-step model and dashed line: 3-step model (partially covered by solid line).

The distributions of intervals between consecutive productions of transcripts, for weak and medium induction are shown in Figure 1. To determine the number and durations of the intermediate rate-limiting steps in initiation, we compare the measured distributions with a sum of *d *exponentially distributed rate-limiting steps. Results are shown in Table 1 for *d *ranging from 1 to 4. The two-step model fits the measurements as well as the models with more steps. The curves that best fit (for d = 1, 2, and 3), along with the measured distributions, are shown in Figure 1.

**Table 1:** Log-likelihood and duration of the steps of the models

Induction	Weak					Medium				
**d**	**Log-likelihood**	**Duration of steps**				**Log-likelihood**	**Duration of steps**			

1	-2029.0	2233				-818	1433			

2	-2000.8	1116	1116			-801	716	716		

3	-2000.5	1099	1099	35		-800	640	640	152	

4	-2000.4	1095	1095	21	21	-800	640	640	152	0

Log-likelihood and duration of the steps of the models with *d *equal to 1 to 4 steps, for weak and medium inductions. There is no implied temporal order for the steps.

The goodness of fit of the models can be assessed by a likelihood-ratio test between pairs of models to reject a null model in favour of the alternative. The results in Table 2 show that, for both weak and medium induction, the single step model is insufficient to explain the measurements, compared to the multi-step models. Further, the tests show that the 2-step model cannot be rejected. This is in agreement with *in vitro *measurements which have shown that both closed and open complex formations are rate-limiting [7,12].

**Table 2:** Likelihood-ratio test between the models

Induction	Weak	Medium
**(d**_**0**_**, d**_**1**_**)**	**p-value**	**p-value**

(1, 2)	3.10 × 10^-14^	3.57 × 10^-9^

(2, 3)	0.4451	0.0955

(3, 4)	0.7186	1

Likelihood-ratio test between pairs of models. Null model is d_0 _step model (where d_0 _equals 1, 2, or 3) while the alternative model is a d_1 _step model (where d_1 _= d_0_+1), in the regimes of weak and medium induction. For p-values smaller than 0.01, it is generally accepted that the null model should be rejected in favour of the alternative. The single step model is insufficient in both regimes.

Since tagged RNA molecules are visible soon after completion, or even while elongating [19], the measured distributions can be explained by transcription initiation being a multi-step process with two or more rate-limiting steps, as in the case of lac UV5 [8]. For that, we need to rule out other alternative explanations, such as the existence of events in elongation, prior to the RNA becoming visible, that significantly affect the intervals between productions of RNAs. The latter explanation can be ruled out as follows. While the mean interval between productions is on the order of 10^3 ^s, elongation only takes tens of seconds (two orders of magnitude smaller) [19]. Events such as long transcriptional pauses during elongation can be ruled out as possible causes since they last 10-100 s [21]. Further, pauses and arrests affect the variance of the distribution, but not the mean [5]. Finally, the eventuality of possible premature terminations can also be ruled out as an explanation for our observations, since they would generate distributions with multiple peaks, centred on multiples of the mean interval between productions.

From all of the above, the events that shape the observed distributions of intervals need to occur during transcription initiation, between the finding of the TSS and initiation of a productive elongation by an RNA polymerase. *In vitro *measurements of the kinetics of initiation of the lar promoter showed that the rate-limiting steps are the formations of the closed and open complexes, which take hundreds of seconds on average under weak and medium induction, while the other steps take only a few seconds [7]. Future experiments, e.g., measurements for genes with the same promoter but an extended sequence, could provide further evidence that elongation does not significantly affect the observed distributions.

From Table 1 and Figure 1, and given the above, we conclude that transcription initiation of the lar promoter, in the regimes of weak and medium inductions, has at least two rate-limiting steps. We note that steps smaller than 60 s are not considered since the interval between consecutive measurements is 60 s. The 150 s step inferred from the measurements under medium induction is not considered significant as well, since it is not detected for weak induction, where all steps are likely to be of equal or greater duration than under medium induction, and because its inclusion does not significantly improve the fit of the model to the data.

Finally, we compared our measurements with previous *in vitro *measurements of the kinetics of the lar promoter [7]. As mentioned, in [7] it was reported that, *in vitro*, when no inducers were present, the mean of intervals between RNA productions events is ~6000 s while for full induction it is ~50 s (~100-fold change). In our *in vivo *measurements, the mean of the intervals were 2233 s for weak induction and 1400 s for medium induction.

Under maximum induction, we observed a production rate of approximately 4 RNA/h per cell. However, this rate of production was only observed if the cells are kept in liquid culture until the moment when they are imaged (see Additional File 2 for details). Due to this, it is not possible to measure its intervals from time series of measurements of individual cells as for the regimes of weak and medium induction. It is only possible to estimate that the intervals have a mean duration of 900 s. Further, by observing cells extracted from the liquid culture at different moments following induction, we found no indication that there may be any significant difference in the dynamics of RNA production in comparison to the regimes of weak and medium induction, apart from the higher mean rate.

Aside from the regime of full induction, the mean intervals measured *in vivo *are of the same order of magnitude as the *in vitro *measurements. This indicates that, *in vivo*, at 24C, the increase in the rate of transcript production due to inducers is, in general, smaller than *in vitro *[7]. This difference is larger for the regime of full induction which could be due to several factors. First, our measurements were made at 24C, rather than 37C, which ought to limit the increase in expression with induction. To test this, we measured mean RNA production at 37C, 1 h following induction. Production increased only by a small amount compared to 24C conditions, indicating that temperature is not the only limiting factor. Another possible cause of the observed divergence between *in vitro *and *in vivo *measurements in full induction is that, *in vivo*, the number of polymerases and other molecules involved in transcription is limited.

## Discussion

Information on the kinetics of the intermediate steps of the multi-step process of transcription initiation in prokaryotes has been limited so far to mean values in *in vitro *conditions, for a limited set of promoters, their mutants [7,12]. Based on the recent development of fluorescence tagging methods for RNA molecules [18,22], combined with statistical analyses, these measurements can now be performed *in vivo*, at the single event level. Understanding the *in vivo *kinetics of transcription initiation is fundamental to understand gene expression regulation. This is necessary to understand dynamics and structure of genetic networks since most of the regulation of RNA and protein numbers in cells occurs at the level of transcription initiation [3].

From the distribution of intervals between consecutive transcription events under the control of the lar promoter in the regimes of weak and medium induction, we inferred the number and duration of the rate-limiting steps in initiation. In both regimes, two rate-limiting steps with approximately equal duration were identified. Their durations were found to be longer, but of the same order of magnitude as the *in vitro *measurements under similar conditions [7,8]. This is expected given the optimal conditions in the *in vitro *measurements, such as controlled temperature, well-stirred environment, and an overabundance of all necessary molecules.

The measurements in the regimes of weak and medium induction reflect the activity of the promoter, which is regulated by the repressors (LacI and AraC) and activators (IPTG and Arabinose), since the binding and unbinding of these molecules to the promoter is a process whose speed is orders of magnitude faster than the process of initiation. Due to this, the intervals between consecutive productions of RNA molecules reflect the kinetics of the promoter, rather than the binding and unbinding dynamics of these molecules [23-26]. The increase in abundance of the inducers causes the intervals between transcription events to differ due to a change in the kinetics of open and closed complex formations [7,8,10-12,15,27]. Our results allow us to conclude that, *in vivo*, IPTG and Arabinose affect the expected duration of both the closed and the open complex formations of the lar promoter, and that both steps are rate-limiting.

It is known from studies of models of gene expression [17] that two exponentially distributed rate-limiting steps in initiation will lead to smaller fluctuations in RNA numbers than when there is only one rate-limiting step. We therefore expect a smaller variance in the RNA numbers produced from the lar promoter than if transcription was a Poisson-like process. Other promoters are known to have much stronger activity under full induction. It may be that there are fewer rate-limiting steps in these cases. This may be the case of the lac promoter, which exhibits Poissonian RNA statistics [28].

Recently, the mRNA copy-number statistics of various promoters were studied in *E. coli *using single-molecule fluorescence *in situ *hybridization (FISH) [29]. A model of transcript production was assumed that includes a two-state promoter (active or inactive), followed by a step associated to transcription initiation of the active promoter. Compared to ours, this model has qualitatively different dynamics of RNA production. This difference is visible by comparing the distributions of intervals between consecutive productions of RNAs (Figure 1 and Additional file 2, Supplementary Figure 4).

Our measurements for the lar promoter favour the model proposed here. However, to account for more complex repression and activation mechanisms that other promoters may have, our model may need to relax the assumption of the exponential duration of the closed complex formation.

Perhaps the most intriguing result here reported is that the inferred time scales of the two rate limiting steps are identical for both induction regimes. It is of interest to speculate whether this is due to some unknown artefact of the inference method or is representative of the real kinetics of transcription initiation of the lar promoter. As noted, we verified that our method of inference reliably distinguishes the duration of each step when they differ by ~25% or more in duration, from 200 intervals sampled from a model of gene expression. However, for smaller differences, the solution is biased towards inferring steps with identical durations, for unknown reasons. Given this, we believe that the inference method is biased towards identical values when and only when the two steps are similar in duration, resulting in a gamma distribution.

In vitro measurements also suggest that, while not identical, the two steps are similar in duration for both weak and medium inductions [7]. This result, in conjunction with the analysis of the simulated data, supports the conclusion that, in vivo, the two steps are similar in duration, under weak and under medium inductions. This in turn suggests that the timescales of the two steps are coupled in the lar promoter. Previous studies suggest that this coupling is likely a consequence of the effects of Arabinose on the dynamics of initiation of this promoter as its concentration affects both open and closed complex formation [7].

Finally, the method used to fit the measured distributions cannot determine the order of the rate-limiting steps. Further measurements and analysis will be required to do so. Nevertheless, our results are informative of the *in vivo *dynamics of events that occur in transcription initiation of the lar promoter and the methodology proposed is applicable to study the kinetics of other promoters, natural or artificial. Further, the results suggest that the duration of the events during transcription initiation, such as the open complex formation, have effects on the dynamics of genetic networks in prokaryotes [27,30], since most genes express rarely during a cell's lifetime [1,4].

## Conclusions

The intermediate steps of transcription initiation are a key regulator of the dynamics of RNA production under the control of the lar promoter in the regimes of weak and medium induction. Since transcription initiation in this promoter has at least two rate-limiting steps, the fluctuations in RNA numbers will be weaker than if there was only one rate-limiting step with the same total mean duration, exponentially distributed. Consequently, cell-to-cell diversity in RNA numbers will also be smaller.

Since most genes in *E. coli *express rarely during a cell's lifetime, and their timely expression is important to many cellular processes, fluctuations in some RNA levels may be damped by a mechanism similar to the one observed here, especially within essential genes. To determine if this is the case, we must characterize the dynamics of the promoters in *E. coli *using similar methods to those proposed here. The methodology used to obtain the distributions of intervals between transcription events, used at the genome-wide scale, promises to provide new insight on the dynamics of gene expression, cell-to-cell diversity in RNA numbers, and consequent phenotypic diversity in bacteria.

## Methods

### Expressing mRNA tagged with MS2d-GFP fusion protein in *E. coli *DH5α-PRO

The method of RNA detection and quantification was proposed in [22] and characterized in E. coli DH5α-PRO [19]. It exploits the ability of bacteriophage MS2 coat protein to tightly bind specific RNA sequences. High resolution detection of single RNA transcripts with 96 tandem repeats of the MS2 binding sites was demonstrated in *E. coli *by using dimeric MS2d fused to GFPmut3 (MS2d-GFP fusion protein) as a detection tag [18]. The method uses the controlled expression of two genetic constructs: a medium-copy vector that expresses MS2d-GFP fused protein, whose promoter (tetO1) is regulated by tetracycline repressor, and a single copy F-based vector, with a lac/ara promoter controlling the production of the transcript target, mRFP1 followed by a 96 MS2 binding site array. Constructs were generously provided by Ido Golding (University of Illinois).

Cells with both MS2d-GFP and transcript target plasmids were grown in Miller LB medium, supplemented by antibiotics according to the specific plasmids. For full induction of protein and RNA, cells were grown in overnight at 37°C with aeration, diluted into fresh medium to maintain exponential growth until reaching an optical density of OD600 ≈ 0.3-0.5. Inducer aTc (100 ng/ml) was added to get full induction of MS2d-GFP production. Approximately 60 min incubation allows sufficient production for RNA detection. After, expression of target RNA is induced (see below).

Following induction, cells are placed on a microscopic slide between a cover slip and 0.8% LB-agarose gel pad set, and visualized by fluorescence microscopy, using a Nikon Eclipse (TE2000-U, Nikon, Tokyo, Japan) inverted C1 confocal laser-scanning system with a 100× Apo TIRF (1.49 NA, oil) objective. GFP fluorescence is measured using a 488 nm laser (Melles-Griot) and a 515/30 nm detection filter. Images of cells are taken from each slide using C1 with Nikon software EZ-C1, approximately 7 min after induction, one per minute, for approximately 2 hours. Measurements under the microscope were made at room temperature (~ 24°C).

Maximum induction of target RNA is achieved with 1 mM of IPTG and 6.7 mM of arabinose [18]. Besides maximum induction, in one case we induced using 5% of the concentrations needed for maximum induction (weak induction), and in another with 15% (medium induction). At maximum induction we observed approximately 4 RNA/cell/hour, in agreement with previous reports and qPCR measurements [18].

We measured the relative changes in mean mRNA numbers with induction strength with quantitative real time PCR. Target RNA was induced with low and high concentrations of inducers. From isolated RNA, complementary DNA was prepared and used for expression analysis [18]. 16S rRNA was used as an internal control. The Livak method [31] was used to confirm the relative gene expression changes. The following primer pairs were used to amplify the mRFP1 region of the target RNA:

Forward: 5' TAC GAC GCC GAG GTC AAG 3'

Reverse: 5' TTG TGG GAG GTG ATG TCC A 3'

and for 16S rRNA:

Forward: 5'CGT CAG CTC GTG TTG TGA A 3'

Reverse: 5' GGA CCG CTG GCA ACA AAG 3'

For details and results of qPCR measurements see Additional File 2.

### Segmentation of cells, MS2-GFP-RNA spots in cells, RNA molecules from spots, and intervals between transcription events

We detect cells from raw images as in [32]. This method divides a greyscale image in three classes: background, cell border and cell region. It then exploits an iterative cell segmentation process that identifies and segments clumped cells based on size and edge information. To avoid degradation of performance of detection in regions where cells are clumped we apply a threshold based on cell size and discard cells whose size goes beyond the threshold.

**Figure 2:**
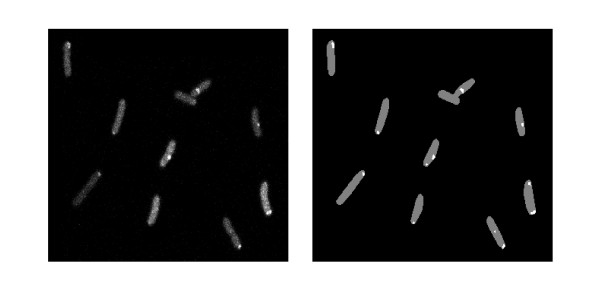
**MS2d-GFP-tagged RNA molecules in E. coli cells**. Unprocessed image of MS2d-GFP-tagged RNA molecules in *E. coli *cells (left) and the corresponding segmented image showing detected cells (grey) and MS2d-GFP-RNA spots (white) within (right).

The automatic spot detection method segments the MS2d-GFP-RNA spots with the kernel density estimation method for spot detection as in [33]. This method estimates the probability density function over the image from local information, and processes the image by filtering it with a desired kernel. We used a Gaussian kernel and then applied Otsu's thresholding method [34] to segment MS2d-GFP-RNA spots from the kernel density estimated image, highlighting the spots (Figure 2). Finally, the number of RNA molecules in each spot was quantified by normalizing the MS2d-GFP-RNA spot intensity distribution as in [18]. This approach, here named the "slicing approach", consists of estimating the number of tagged transcripts in the cell by dividing a spot's intensity by the intensity of the first peak in the histogram of spot intensities (Additional file 2, supplementary figure 3). An example of a distribution of spot intensities, obtained from the images of multiple cells is shown in Additional File 2.

By performing this analysis for each frame, it is possible to determine when new RNA molecules appear in the cell. From that, we calculate intervals between the productions of consecutive RNAs in individual cells. For a detailed description of this analysis, as well as examples, refer to [18] and Additional files.

Finally, we only count intervals between consecutive RNA molecules which are produced in the same cell. If a cell division occurs, the interval between the last RNA produced in the mother cell and the first RNA produced in a daughter cell is not included in the counts of intervals between consecutive RNA molecules.

### Fitting the model to a d-step model, each step with an exponentially distributed duration

Given the distribution of time intervals between consecutive transcription events, obtained from multiple cells subject to the same induction, it is possible to determine the maximum likelihood fit of a model with *d *statistically independent steps, whose time lengths each follow and exponential distribution. For such a d-step model with parameters μ = [μ_1_, μ_2,_..., μ_d_], and given N measured intervals between transcription events, Δt_k_, where k goes from 1 to N, the log-likelihood is:

(3) $$L\left(\mu \right)=\overset{N}{\underset{k=1}{\sum }}\log\pi_{d}\left(\Delta t_{k};\mu \right)$$

where π_d _is the probability density function for a sum of d exponential random variables with means μ_d_. The probability density function for the sum can be found by the convolution of the probability density functions of the individual exponential random variables. The density functions for d = 1,..3 are:

(4) $$\pi_{1}\left(\Delta t_{k};\mu_{1}\right)=\frac{e^{\frac{-x}{\mu_{1}}}}{\mu_{1}}$$

(5) $$\pi_{2}\left(\Delta t_{k};\mu_{1},\mu_{2}\right)=\frac{e^{\frac{-x}{\mu_{1}}}}{\mu_{1}-\mu_{2}}+\frac{e^{\frac{-x}{\mu_{2}}}}{\mu_{2}-\mu_{1}}$$

(6) $$\begin{array}{c} \pi_{3}\left(\Delta t_{k};\mu_{1},\mu_{2},\mu_{3}\right)=\frac{\mu_{1}e^{\frac{-x}{\mu_{1}}}}{\left(\mu_{1}-\mu_{2}\right)\left(\mu_{1}-\mu_{3}\right)}\\ \;+\frac{\mu_{2}e^{\frac{-x}{\mu_{2}}}}{\left(\mu_{2}-\mu_{1}\right)\left(\mu_{2}-\mu_{3}\right)}+\frac{\mu_{3}e^{\frac{-x}{\mu_{3}}}}{\left(\mu_{3}-\mu_{1}\right)\left(\mu_{3}-\mu_{2}\right)} \end{array}$$

The values of μ = [μ_1_, μ_2 _... μ_d_] are the expected means and standard deviations of the durations of each of the steps composing the intervals between production events. We use this procedure to find the values of μ that provide the highest log-likelihood for d = 1,...,4. No significant improvement of fit was observed for values of d > 2 (Table 1).

We note that the singularities of the probability density functions, formulas (5) and (6), were not problematic since the maximum likelihood estimate of the μ's differed from the second decimal onward. Furthermore, the singularities can be removed. For example, in (5), if μ_1 _= μ_2_, the singularity can be removed by various means (e.g. L'Hôpital rule), so that π_2 _equals the density function of the gamma distribution with parameters k = 2 and θ = μ_1 _= μ_2_.

The goodness of fit of the models can be assessed by comparison. For that, we perform a likelihood-ratio test between pairs of models to reject a null model in favour of the alternative. Finally, we verified that the method reliably distinguishes the duration of each step, when they differ by ~25% in duration, from 200 intervals sampled from a model of gene expression.

## Authors' contributions

ASR conceived the manuscript. MK executed the experiments. HM, AH, JLP and AG executed the analytical studies. OYH participated in the planning with ASR. ASR wrote most of the manuscript. All authors contributed in the writing, read and approved the final manuscript.

## Supplementary Material

Additional file 1 **Example of a movie generated from temporal images of a cell**. Images were taken approximately 7 min after induction, one every minute, for approximately 2 hours. The cell was induced with 0.01 mM of IPTG and 0.067 mM of Arabinose. The cell identification number and the time (s) when the frame was captured are shown in the top right and left corners, respectively. Click here for file

Additional file 2 **Supplementary information**. Supplementary information: qPCR analysis of the target RNA; image analysis and cell segmentation, detection and counting of mRNA in cells; analyses of the intervals between production events assuming an ON-OFF mechanism of RNA production; measurements of RNA numbers under full induction. Click here for file
