# Supplementary material for: In vivo kinetics of transcription initiation of the lar promoter in Escherichia coli. Evidence for a sequential mechanism with two rate-limiting steps
Source: BMC Syst Biol. 2011 Sep 25;5:149. doi: 10.1186/1752-0509-5-149 (PMC3191489; doi:10.1186/1752-0509-5-149)
Supplement: Additional file 2 — Supplementary information. Supplementary information: qPCR analysis of the target RNA; image analysis and cell segmentation, detection and counting of mRNA in cells; analyses of the intervals between production events assuming an ON-OFF mechanism of RNA production; measurements of RNA numbers under full induction. [file 1752-0509-5-149-S2.PDF]

<u>Supplementary File 2:</u> In vivo kinetics of transcription initiation of the lar promoter in Escherichia coli. Evidence for a sequential mechanism with two rate-limiting steps.

Authors: Meenakshisundaram Kandhavelu, Henrik Mannerström, Abhishekh Gupta, Antti Häkkinen, Jason Lloyd-Price, Olli Yli-Harja and Andre S Ribeiro

---

I. qPCR analysis of the target RNA:

Gene expression was induced as described in the material and methods section in the main document. Total RNA was isolated using RNeasy kit (Qiagen) according to the manufactures' instructions. DNaseI treatment was performed before cDNA synthesis. Maxima® First Strand cDNA Synthesis Kit was used to make cDNA (Fermentas). Primers for mRFP1, 16S rRNA were used to amplify the desired length. Real time PCR experiment was performed using Maxima^{TM} SYBR Green qPCR Master Mix in a Biorad MiniOpticon Real time PCR system. The following thermal cycling protocol was used: 35 cycles of 94˚C for 15 s, 54˚C for 30 s, and 72˚C for 30 s for each cDNA replicate. RT–qPCR reactions were performed by using primers (mRFP1-Fw: 5' TAC GAC GCC GAG GTC AAG 3'; mRFP1-Rv: 5' TTG TGG GAG GTG ATG TCC A 3') for the target gene and primers for housekeeping genes (16S rRNA-Fw: 5'CGT CAG CTC GTG TTG TGA A 3'; 16S rRNA-Rv: 5' GGA CCG CTG GCA ACA AAG 3') as an internal reference. Replicate samples were used to quantify the gene expression in the final volume of 20µL reaction. Two independent sets of experiments were performed with primer efficiencies of these reactions not less than 90%. CFX Manager^{TM} Software was used to obtain the amplified gene expression pattern for the target and 16S rRNA housekeeping gene and then the Livak method (for reference see main document) was used to confirm the relative gene expression changes.

Results are shown in supplementary figure 1, and the change in RNA relative expression level with induction (from weak to medium) is in accordance with the changes measured in live cells under confocal microscopy (reported in the main document).

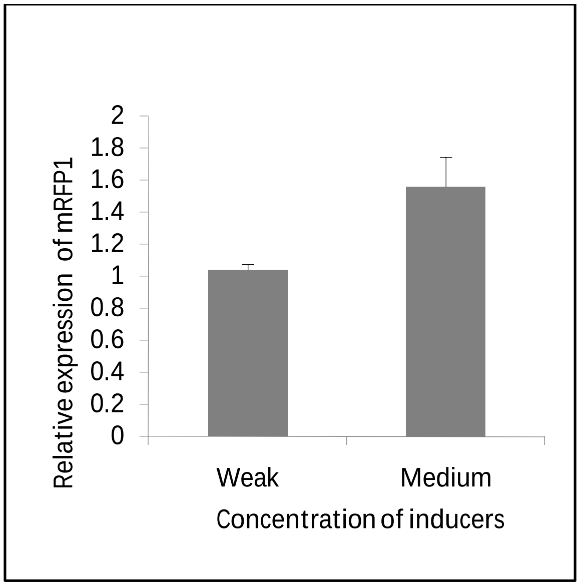

Supplementary figure 1: **mRFP1 gene expression analysis**: The relative changes in mean mRNA numbers with induction strength were measured with quantitative real time PCR. Target RNA was induced with weak and medium concentrations of the inducers. We observe that mRFP1 gene expression in the medium induction case is 1.6 times that of the weak induction case, as calculated by the Livak method. Error bars indicate the statistical significance between two independent measures of real time experiments.

II. Image analysis and cell segmentation. Extraction of the number of RNAs in each cell at each moment.

Once the images of the cells are acquired by confocal microscopy (supplementary figure 2), we analyze them in a semi-automated fashion to detect and count the target RNA. This process starts by segmenting the cells as well as the RNA spots within. From there, the distribution of spot intensities from cells in each image can be obtained.

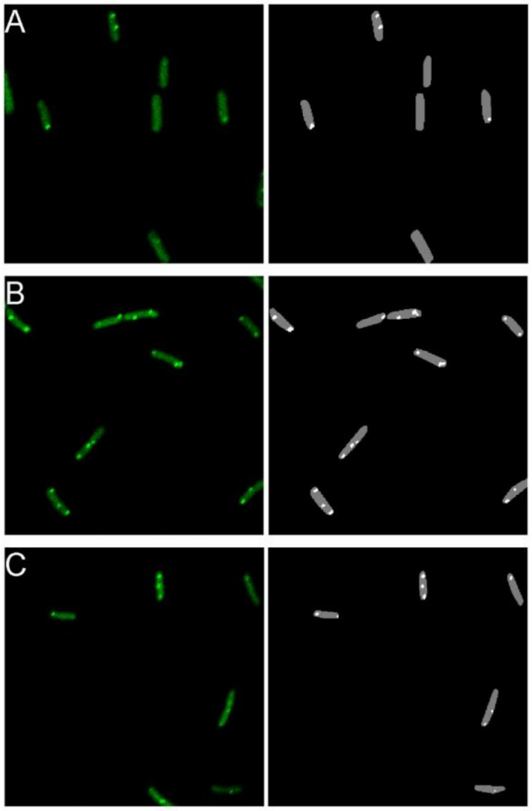

Supplementary figure 2: Examples of original and segmented images of cells and spots within: A-C. Examples of images taken by confocal microscope of MS2-GFP-tagged RNA molecules in *E. coli* cells. Unprocessed image (left) and the corresponding segmented image (right) showing the detected cells in grey and the spots in white.

The number of tagged transcripts in a cell can be estimated by dividing a spot's intensity by the intensity of the first peak in the histogram of spots intensities (supplementary figure 3). Relevantly, at very low induction levels, only one peak is detected, and it corresponds to the intensity of a single tagged RNA molecule (see below, as well as the main document and ref. [12] within). According to this method, here referred to as the "slicing approach", the estimated number of RNA molecules per spot is equal to its total fluorescence, normalized by the intensity of a single tagged mRNA molecule and rounded to the nearest integer. The intensity of one RNA molecule equals the intensity of the first peak, as calculated from the tagged mRNA molecules (supplementary figure 2).

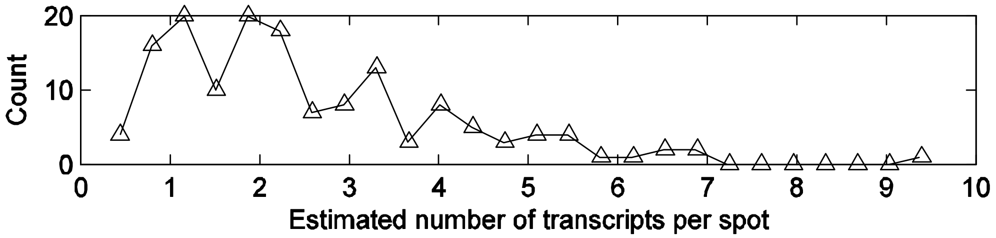

Supplementary figure 3: Counting of mRNA in cells: Distribution of number of spots and their intensity in a cell population. The estimated number of RNA molecules per spot is equal to its total fluorescence normalized by the intensity of a single tagged mRNA molecule (intensity of the first peak).

For a time series measurement, the process of segmentation is made for each individual image, independently. Then, the overall distribution of spot intensity is generated, obtained from all cells at each time point. From that, the number of RNA molecules in each cell, at each time point, can be obtained. By counting the number of RNAs in each cell at each moment, it is

possible to determine when a new RNA appears and, thus, the time between the appearance of consecutive RNA molecules in individual cells. From that, we can generate distributions of intervals between consecutive transcription events for each cell, in multiple cells subject to the same level of induction of the target gene.

III. Time intervals between production events assuming an ON-OFF mechanism of RNA production.

Our model of transcription initiation, which is derived from the works of McClure and Lutz (refs. [5] and [7] in the main document), differs from the model proposed in ref. [33] of the main document. In [33], the mRNA copy-number statistics of several promoters were studied in *E. coli* using single-molecule fluorescence in situ hybridization (FISH). A model of transcript production was assumed that includes a two-state promoter (available or not for transcription), followed by a step associated to initiation on the active promoter. This model has a different dynamics of RNA production and consequent cell to cell diversity in RNA numbers than ours. This is visible by comparing the distributions of intervals between consecutive productions of RNA molecules (supplementary figure 4 and Fig. 1 in main document). The model of RNA transcription in ref. [33] consists of the following set of reactions:

$$Off \underset{k_{off}}{\overset{k_{on}}{\rightleftarrows}} On \xrightarrow{k_{TX}} mRNA \qquad (1)$$

In reaction (1), 'Off' is the promoter in the OFF state, 'On' is the promoter in the ON state, and mRNA is a complete RNA molecule. The RNA level is determined by the rate constants, namely, by k_{on}, the rate of switching to the ON state; by k_{off}, which sets the rate of

switching from the ON to the OFF state; and by k_{TX}, which is the rate of producing RNA molecules while in the ON state. Changes in the dynamics of RNA numbers occur if one modifies any of the kinetic parameters controlling RNA production.

It is possible to show that, for any value of the kinetic rates of RNA production, this model always produces a coefficient of variation of time intervals between consecutive RNA molecules (standard deviation over the mean) that is greater than 1. Let T_R be the distribution of intervals between the productions of two consecutive RNA molecules. The distribution of T_R is determined by the three rates above. It is possible to show that the mean and variance of this distribution are given by:

$$E\left[T_R\right]=\frac{1}{k_{TX}}\left(1+\frac{k_{off}}{k_{on}}\right) \quad (2)$$

$$Var\left[T_R\right]=\frac{1}{k_{TX}^2}\left(1+\left(2+2\frac{k_{TX}}{k_{on}}+\frac{k_{off}}{k_{on}}\right)\frac{k_{off}}{k_{on}}\right) \quad (3)$$

From (2) and (3), one can calculate the coefficient of variation. By evaluating the ratio between (3) and the square of (2), it is possible to see that it is always greater than 1.

The distribution of T_R itself (supplementary figure 4) resembles an exponential distribution with a heavy tail (thus it differs significantly from our measured distributions in the main document). Since this distribution's mode is at 0 and it has many long intervals where no transcription occurs, the difference between this model and ours is apparent. Sampled distributions of this model are shown in supplementary figure 4.

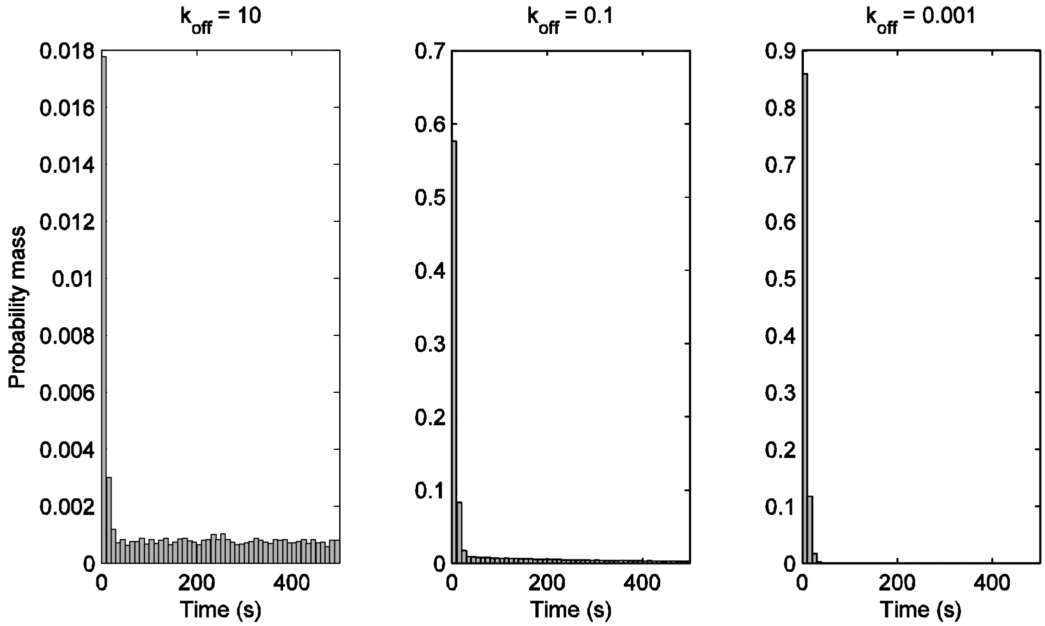

Supplementary figure 4: Probability distribution of intervals between consecutive RNA molecules for varying k_{off}. These distributions are derived from the model described by supplementary equation (1) for three values of k_{off}. Values of k_{tx} and k_{on} are set to 0.2 and 0.004 s^{-1}, respectively (as in ref. [33] in main document). Binning is set to 10.

Finally, it is noted that under certain conditions, the two models produce similar dynamics of transcription. If, in our model, the open complex formation and clearance are short in duration and if in model (1) the ratio between k_{off} and k_{on} is small, than the two models have Poisson-like dynamics. Another way is to relax the assumption that the duration of the first step in our model follows an exponential distribution, and in this case the condition on ratio between k_{off} and k_{on} is no longer necessary.

IV. Measurements under full induction:

We were unable to observe full induction in time series measurements. To observe full induction, cells need to be kept in liquid and optimal growth condition just prior to measurement.

These measurements thus consist of images of cells taken 1 hour after full induction (1mM IPTG and 6.7 mM Arabinose). The cells are kept in liquid culture until the moment of measurement. We measured 4.04 RNA/cell from a population of 186 cells. Two replicas obtained in independent experiments confirmed this result. These results agree with previous reports (referenced in the main document). An example image of cells of such populations is shown in Supplementary Fig. 5.

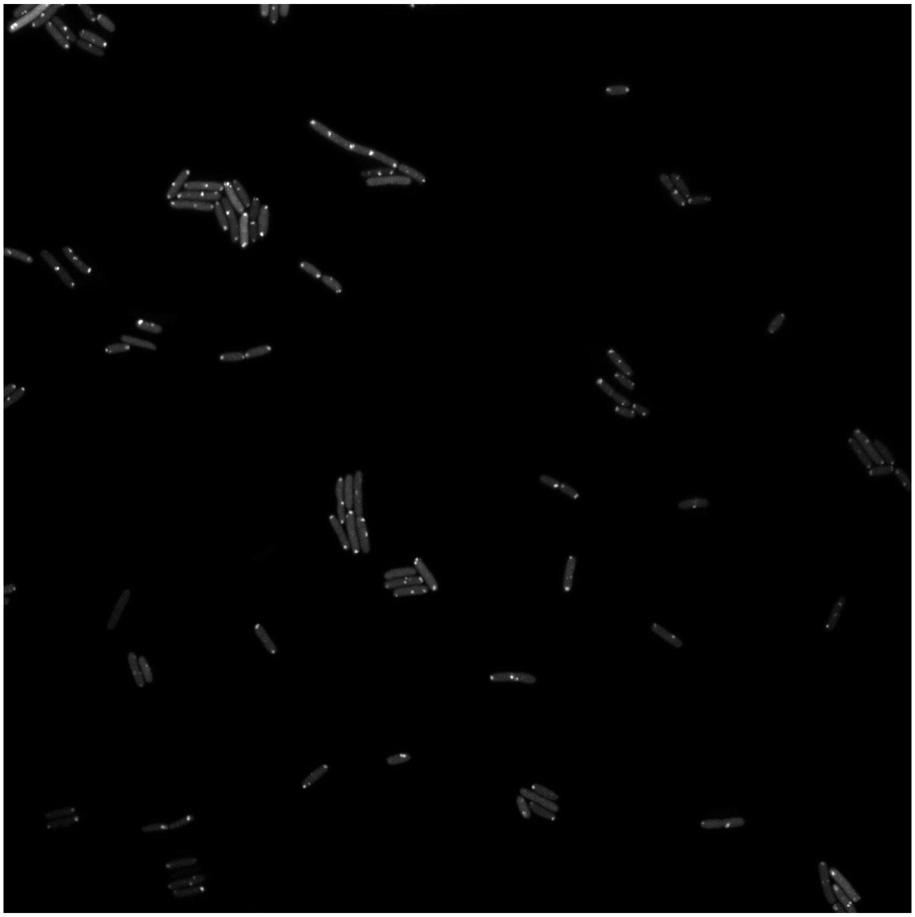

Supplementary figure 5: Detection of mRNA in cells of a population 1 h following induction. The cells are subject to full induction and kept in liquid culture until the moment of measurement. Most spots correspond to more than a single RNA molecule and, thus, the number of RNA molecules per cell can only be counted following the slicing approach.
